# Economic impact of patients with medical evacuation in remote islands: a case study in Matsu Islands

**DOI:** 10.3389/fpubh.2025.1542172

**Published:** 2025-05-30

**Authors:** Ping-Hsuan Hsieh, Bo-Xiang Shi, Chung-Yu Lai, Yin-Chung Chen, Chin Lin, Olivia Wu, Sy-Jou Chen, Shih-Hung Tsai

**Affiliations:** ^1^School of Pharmacy, National Defense Medical Center, Taipei, Taiwan; ^2^Graduate Institute of Aerospace and Undersea Medicine, National Defense Medical Center, Taipei, Taiwan; ^3^Combat and Disaster Casualty Care Training Center, National Defense Medical Center, Taipei, Taiwan; ^4^Department of Emergency Medicine, Tri-Service General, Hospital National Defense Medical Center, Taipei, Taiwan; ^5^School of Medicine, National Defense Medical Center, Taipei, Taiwan; ^6^Health Economics and Health Technology Assessment, School of Health and Wellbeing, University of Glasgow, Glasgow, United Kingdom; ^7^Department of Physiology and Biophysics, Graduate Institute of Physiology, National Defense Medical Center, Taipei, Taiwan; ^8^Taichung Armed Forces General Hospital, Taichung, Taiwan

**Keywords:** economic burden, air medical evacuation, remote island, cost analysis, medical evacuation

## Abstract

**Objective:**

Air medical evacuation for residents of remote islands is expensive yet essential for addressing urgent and critical health conditions. This study aims to identify common referral reasons and quantify the economic impact of such services, with a focus on the potential benefits of implementing preventive medicine and telemedicine to improve medical care accessibility and coverage in these regions.

**Methods:**

We conducted a retrospective analysis of adult patients who traveled from the Matsu Islands to the Taiwan mainland between January 2016 and June 2022 and divided them into two groups: Emergency Air Medical Transport (EAMT) and non-EAMT. We included both direct medical and non-medical costs, and indirect costs measured by productivity loss due to health conditions. A generalized linear model adjusted for age and gender was employed to estimate average costs per patient.

**Results:**

Data were available for 423 participants, with 136 in the EAMT group and 287 in the non-medical evacuation group. The average direct costs were significantly higher in the EAMT group ($12,067, 95% confidence interval [CI] 9,592–15,181) compared to the non-EAMT group ($5,540, 95% CI 4,645–6,608). Transportation costs made up the largest portion of these direct costs, particularly for those requiring EAMT services. The most common referral reasons for EAMT were cardiovascular diseases (27.2%), followed by injuries (21.3%) and general conditions (15.4%). Across all referral reasons, the EAMT group consistently incurred higher average costs compared to the non-EAMT group, with fractures resulting in the highest costs ($21,342, 95% CI 13,794–33,019).

**Conclusion:**

The findings highlight the significant financial burden of medical evacuation services in remote islands, particularly for cardiovascular conditions, injuries, and fractures. These results emphasize the need for targeted preventive measures and improved healthcare access to reduce both economic impact and health risks, providing a basis for further cost-effectiveness analysis of future interventions.

## Introduction

Remote islands face difficulties due to a lack of accessibility, manpower, and facilities, stemming from the uneven distribution of medical resources. The delivery of health services to these areas is particularly challenging because of their relatively small populations and inaccessible locations (1, 2). These difficulties are exacerbated by limited funding and a shortage of healthcare professionals in many countries (3, 4). The scarcity of healthcare resources also influences health-seeking behaviors, which differ significantly between residents in rural and urban areas (4, 5). Residents of remote islands have fewer options and often need to rely on the same healthcare provider regardless of the type and severity of the illness. The lower accessibility of healthcare providers on remote islands further reduces residents’ willingness to seek medical care, potentially leading to the worsening of diseases, or even life-threatening situations (4).

The Matsu Islands, part of Taiwan’s offshore territories, struggle to deliver comprehensive healthcare to their 12,000 residents due to limited facilities: one hospital and five clinics. As of 2021, the local healthcare workforce consisted of 12 physicians, supported by a small team of nurses, paramedics, and allied health professionals, with only 43 inpatient beds available. This shortage of personnel and resources presents significant challenges in ensuring timely and adequate medical care (6). To address the shortage of emergency and intensive care resources, the Ministry of Health and Welfare (MOHW) in Taiwan introduced the Emergency Air Medical Transport (EAMT) service and its approval mechanism in 2002. The National Aeromedical Approval Center (NAAC), under MOHW, manages applications, evaluations, and coordination for EAMT. A civilian aircraft dedicated to EAMT has been deployed to improve response times, particularly for offshore areas.

The EAMT approval mechanism ensures timely and appropriate medical care when local healthcare capacity is insufficient. Approval requires confirmation that the receiving hospital can provide the necessary treatment and that the transport will be carried out with appropriate medical equipment and trained personnel. EAMT is typically reserved for life-threatening conditions such as severe trauma, unstable vital signs, or specific medical emergencies. Examples include patients with severe burns, respiratory complications following drowning, high-risk labor, acute stroke, or life-threatening cardiac conditions such as aortic dissection (7).

Debates regarding the potential less cost-efficient implementation of advanced healthcare facilities versus providing risky and yet still costly urgent medical evacuation currently exist (8). The implementation of preventive medicine and telemedicine have been proposed to improve the accessibility and coverage of medical care in remote islands (9, 10). No study has yet estimated the economic impact of medical evacuation in remote islands. This study aims to use Matsu as an example to explore the economic burden in patients with medical evacuation and those seeking healthcare on their own, distinguishing between the referral reasons. Understanding the economic impact based on common referral reasons can provide valuable insights for policymaking, resource allocation, and implementing preventive measures for remote communities.

## Methods

### Database source

We conducted a retrospective analysis on adult patients (aged ≥18 years) who traveled from the Matsu islands to Tri-Service General Hospital (TSGH), which is the primary hospital for handling patients medically evacuated from Matsu to Taiwan. Medical records were reviewed from the Emergency Department. Each patient’s visit was de-identified and coded by a unique study number. The study received approval from the institutional review board for human investigations at TSGH, which also waived the requirement for informed consent. Data collection involved hospital records from TSGH during January 1, 2016, to June 30, 2022. The collected data included patient demographics, main diagnoses, dates of arrival, and discharge. Health resource utilization was obtained through matching hospital records. Patients from Matsu were divided into two groups: Emergency Air Medical Transport (EAMT) and non-EAMT. The EAMT group included patients who received emergency air medical evacuation services due to urgent clinical needs. The non-EAMT group consisted of patients transferred through standard (non-emergency) means. Further details on the criteria of EAMT are provided in Appendix I. Those transferred to TSGH via the EAMT service were identified by matching the administrative records provided by the Matsu government. The EAMT group comprised patients who met the medical evacuation criteria and were transported by helicopter. In contrast, the non-medical evacuation group included individuals who sought hospital care without referral by the local government in Matsu. Reasons for referral were determined based on the primary diagnosis at discharge, classified according to the International Statistical Classification of Diseases and Related Health Problems, 10th Revision (ICD-10) codes. ICD-10 codes were grouped into organ system/disease categories by the first three digits.

### Cost analysis

The cost analysis includes both direct and indirect costs during the medical visit from Matsu to Taipei from a societal perspective. Direct costs, incurred by both the healthcare system and individuals, are categorized into medical and non-medical components. Medical costs encompass health resource utilization such as medication, hospitalization, outpatient attendance, and diagnostic examinations, all of which were thoroughly documented in hospital records. Non-medical costs involve resources such as transportation to the healthcare provider and informal care provided by caregivers. For those requiring EAMT services, transportation costs were calculated as $7,231, representing the current emergency transfer model with the AW-169 helicopter. For the non-medical evacuation group, costs were based on the price for a commercial flight from Matsu to Taipei ($154). It is assumed that each patient would require a caregiver; therefore, costs for informal care were measured by the length of hospitalization and multiplied by the average wage ($110 daily).

Indirect costs arise from absenteeism and presenteeism. However, since the database lacks information on presenteeism, these costs were primarily estimated based on the length of hospitalization. Therefore, indirect costs were estimated by multiplying the length of hospitalization by age- and gender-specific average wages. The human capital approach (HCA) was applied to reflect lost productive potential (11–13), with wages obtained from data published by the Taiwan government in 2022 (14).

### Statistical analysis

Continuous data were reported as mean and standard deviation. For variables not normally distributed, data were presented as median and interquartile range. Comparisons of continuous data between patients with and without EAMT services were performed using unpaired Student’s *t*-tests or Dunn’s *post hoc* tests following the Kruskal-Wallis test, where appropriate. Discrete data were described as frequency and percent. Given the often right-skewed nature of cost data, we employed a generalized linear regression model (GLM) with a log-link function and a gamma distribution to achieve data symmetry (15, 16). For the GLM model, the dependent variable was total medical cost. The primary independent variable was the EAMT group, with the non-EAMT group serving as the reference. The model also adjusted for age and gender, using the youngest age group and female sex as reference categories. All costs were converted to US dollars (currency on 21 April 2023, 1 USD = 32.54 TWD). This statistical analysis was performed using R, version 4.2.2. The level of statistical significance was considered at *p* < 0.05.

## Results

In total, 423 patients were included in the analysis, with 136 in the EAMT group and 287 in the non-medical evacuation group. The annual enrollment of patients in this study is detailed in Supplementary Table 1. Males comprised approximately three-quarters of both groups. Patients in the EAMT group were generally older than those in the non-EAMT group, with mean ages of 57.0 and 48.8 years, respectively (*p* < 0.05). A higher proportion of middle-aged and older adult individuals were in the EAMT group, while the age distribution was more balanced in the non-EAMT group, as shown in Table 1.

**Table 1:** Patient demographics stratified by EAMT and non-EAMT.

Variables	EAMT	Non-EAMT
Patients	136	287
Sex		
Male	99 (72.8%)	219 (76.3%)
Female	37 (27.2%)	68 (23.7%)
Age*		
Mean (SD)	57.0 (20.0)	48.8 (21.6)
Median (IQR)	57 (18–100)	47 (18–109)
Age group		
<24	21 (15.4%)	88 (30.7%)
35–44	10 (7.4%)	33 (11.5%)
45–54	31 (22.8%)	49 (17.1%)
55–64	33 (24.3%)	47 (16.4%)
≥65	41 (30.1%)	70 (24.4%)

EAMT, Emergency Air Medical Transport; IQR, interquartile range; SD, standard deviation.

All patients are grouped according to their primary diagnoses in ICD-10 codes and summarized in Supplementary Table 2. Among those who required EAMT services, cardiovascular diseases and injuries were the most common referral reasons, accounting for 27.2 and 21.3% of patients, respectively. Conversely, general conditions and other symptoms (including fever, fatigue, and shock) and injuries were the most common reasons among residents seeking healthcare on the mainland, representing 23.0 and 22.0% of patients, respectively. Additionally, less acute conditions such as respiratory symptoms and gastrointestinal disorders were also frequent among mainland healthcare seekers, each accounting for 9.4% of the cases (Table 2).

**Table 2:** Common referral reasons stratified by EAMT and non-EAMT.

EAMT (*n* = 136)		Non-EAMT (*n* = 287)	
ICD-10 category	n (%)	ICD-10 category	n (%)
Cardiovascular	37 (27.2%)	General conditions and others	66 (23.0%)
Injury	29 (21.3%)	Injury	63 (22.0%)
General conditions and others	21 (15.4%)	Respiratory	27 (9.4%)
Respiratory	13 (9.6%)	Gastrointestinal	27 (9.4%)
Musculoskeletal	10 (7.4%)	Cardiovascular	21 (7.3%)
Others	26 (19.1%)	Others	83 (28.9%)

*EAMT, Emergency Air Medical Transport; ICD-10, International Classification of Diseases, Tenth Revision, Clinical Modification.

As shown in Table 3, average direct costs were $12,067 (95% CI 9,592–15,181) for EAMT patients and $5,540 (95% CI 4,645–6,608) for non-EAMT patients. The main difference was due to non-medical costs, primarily transportation. Medical costs were $2,153 (95% CI 1,550–2,990) for EAMT patients and $1,311 (95% CI 1,019–1,687) for non-EAMT patients. Non-medical costs were $9,588 (95% CI 8,161–11,266) for EAMT patients and $4,124 (95% CI 3,666–4,595) for non-EAMT patients. Indirect costs were higher for EAMT patients at $730 (95% CI 521–1,024) compared to $467 (95% CI 360–605) for non-EAMT patients, primarily due to longer hospital stays. Consequently, total costs (combining direct and indirect costs) for EAMT patients were approximately twice as high at $12,975 (95% CI 10,253–16,420) compared to $6,049 (95% CI 5,049–7,248) for non-EAMT patients.

**Table 3:** Average cost of patients stratified by EAMT and non-EAMT.

Cost components	EAMT	Non-EAMT
Direct costs^a^	12,067 (9,592–15,181)	5,540 (4,645–6,608)
Medical costs	2,153 (1,550–2,990)	1,311 (1,019–1,687)
Non-medical costs	9,588 (8,161–11,266)	4,124 (3,666–4,595)
Indirect costs	730 (521–1,024)	467 (360–605)
Total costs^b^	12,975 (10,253–16,420)	6,049 (5,049–7,248)

*Results are shown as mean (95% CI).^a^Direct costs were the sum of medical and non-medical costs.^b^Total costs were the sum of direct and indirect costs. EAMT, Emergency Air Medical Transport.

Table 4 shows that musculoskeletal disorders incurred the highest costs for EAMT patients (mostly attributed to fractures), followed by neurological conditions, general conditions, and respiratory diseases. Patterns were similar in both groups, with musculoskeletal and neurological conditions also leading to high costs in the non-EAMT group. Costs for injuries and genitourinary disorders were significantly higher in the EAMT group compared to the non-EAMT group. Overall, EAMT patients experienced substantially higher costs across all conditions, highlighting the financial impact of emergency air medical transport services.

**Table 4:** Estimated cost in the EAMT and non-EAMT groups arranged by total costs.

Referral reason	EAMT		Non-EAMT	
	Estimated cost	n	Estimated cost	n
Musculoskeletal	21,342 (13,794–33,019)	10	9,950 (6,450–15,351)	10
Neurologic	14,371 (7,069–29,219)	3	6,700 (3,314–13,546)	4
General conditions and others	14,235 (11,018–18,390)	21	6,636 (5,378–8,190)	66
Respiratory	13,089 (9,374–18,277)	13	6,103 (4,489–8,296)	27
Cardiovascular	13,077 (9,798–17,452)	37	6,097 (4,513–8,236)	21
Oncology and hematology	12,379 (7,227–21,203)	4	5,771 (3,444–9,671)	15
Infectious	11,917 (7,247–19,596)	3	5,556 (3,489–8,847)	16
Injury*	11,354 (8,862–14,546)	31	5,293 (4,264–6,571)	66
Gastrointestinal	10,832 (7,590–15,460)	8	5,050 (3,657–6,973)	31
Genitourinary*	9,195 (5,241–16,132)	2	4,287 (2,511–7,318)	10
Obstetrics	7,175 (2,816–18,281)	2	3,345 (1,310–8,543)	2

^a^Results are shown as mean (95% CI); ^b^Total costs were the sum of direct and indirect costs.^*^p < 0.05.EAMT, Emergency Air Medical Transport; ICD-10, International Classification of Diseases, Tenth Revision, Clinical Modification.

## Discussion

In the present study, 423 patients from Matsu were included and further divided into EAMT and non-EAMT groups. In both groups, males accounted for the majority, and individuals in the former group were older (57.0 vs. 48.8 years) than those in the latter group. The average total costs for EAMT patients were 2.14 times higher than those in the non-EAMT group ($12,975 vs. $6,049), mostly attributed to costs for transportation. The most common referral reasons for EAMT patients were cardiovascular diseases, injuries, general conditions, respiratory symptoms, and musculoskeletal disorders, with musculoskeletal disorders (primarily due to fractures) incurring the highest costs. Across various referral reasons, EAMT patients experienced substantially higher costs, highlighting the financial impact of emergency air medical transport services.

The average direct costs per patient in the EAMT group were higher than those in the group not requiring medical evacuation, covering medication, hospitalization, and other cost components. This difference suggests that patients requiring EAMT services often face more severe health conditions. Across all referral reasons, the EAMT group consistently incurs higher average costs compared to the non-EAMT group, generally more than double. However, the variability in costs, as indicated by the confidence intervals, suggests that the severity of conditions and the complexity of treatments required for patients needing air transport can vary widely. Notably, the costs associated with musculoskeletal conditions are the highest among all categories, indicating that these cases may require particularly complex and resource-intensive care.

Transportation costs were the predominant direct expense for EAMT patients - significantly higher than the usual cost distribution for various diseases. For comparison, a comprehensive review of cost-of-illness studies for heart failure identified hospitalization as the major cost driver, representing 44–96% of direct costs. A similar trend was observed in patients with spinal cord injuries, where hospitalization costs again constituted the primary financial burden (17, 18). Additionally, an annual contract of $215,120 was established for the civilian aircraft used for EAMT services, though these operational costs were not included in our cost analysis.

Beyond transportation costs, the geographic isolation of remote areas presents challenges in recruiting and retaining healthcare professionals due to unequal access to education and professional support, the necessity of working beyond their usual scope of practice, safety concerns, and adapting to extreme weather conditions (19). Our study pinpointed acute cardiovascular diseases, injuries, and musculoskeletal disorders, specifically bone fractures, as the leading causes for air medical evacuations. Thus, implementing preventative measures could significantly reduce the need for such evacuations. It is vital to focus on individuals displaying early symptoms of circulatory issues, managing disease progression through medications and lifestyle modifications. For example, timely access to percutaneous coronary intervention (PCI) is crucial and can significantly affect the impact of a hospital’s rural location on in-hospital mortality rates in patients with acute myocardial infraction (20). Yet, patients from rural communities are less likely to receive timely PCI, facing higher mortality and readmission rates compared to those in urban areas (21). Prolonged door-to-balloon (D2B) times were identified as predictors of in-hospital death, particularly in rural settings. The disparity in in-hospital mortality between rural and urban patients becomes especially pronounced in cases of severe heart failure with D2B times exceeding 90 min (22).

Fractures were one of the most frequent reasons for patients requiring the EAMT service in the Matsu Islands, incurring the highest costs. Falls, especially among the older adult in rural areas, are the primary cause of emergency room visits, hospitalizations, and deaths associated with hip fractures (23). Furthermore, rural residents experience a higher mortality rate following hip fractures compared to those living in urban areas (24). Research has shown that awareness of fall risk factors among older residents in rural communities can be lacking (25). Therefore, health education initiatives and fall prevention services aimed at improving knowledge, attitudes, and practices regarding fall prevention for the older adult are crucial in mitigating the impact of falls in these populations.

Air medical evacuation, though costly, is crucial for providing urgent medical care on remote islands. The cost-effectiveness analysis by Delgado et al., which compared helicopter emergency medical services (HEMS) to ground transportation for trauma incidents, indicated that HEMS needs to provide at least a 15% mortality reduction or a measurable improvement in long-term disability to compare favorably with other interventions will be considered cost-effective. Therefore, reducing overtriage of patients with minor injury to HEMS would improve its cost-effectiveness (26). Furthermore, Tsai et al. found that physician-assisted pre-flight screening via video telemedicine substantially lowered the rate of unnecessary air medical transports, thus reducing costs (27).

This study has several limitations. First, while the population of the Matsu Islands is approximately 12,000, with 57.7% male and 12.6% aged over 65 years (28), our sample comprised approximately 75% male patients in both the EAMT and non-EAMT groups. This may reflect a higher demand for medical evacuation among men, possibly due to the prevalence of cardiovascular diseases and work-related injuries (29–31). Although our sample does not fully reflect the demographic distribution of the overall population, it likely represents the subset requiring emergency medical services. Additionally, although TSGH is the primary referral center for Matsu residents, 36.9% of patients were transferred to other hospitals, with only 9.7% referred to the second most common destination. This distribution suggests that our data still provides a relevant representation of medical evacuation needs in this region.

Second, referral reasons were classified based on the primary discharge diagnosis to ensure diagnostic accuracy. However, in patients with multiple comorbidities, this approach may have limited our ability to capture the full complexity of their clinical conditions. Third, the lack of information on care received prior to arrival in Taiwan may have led to underestimation of direct medical costs, particularly for patients who received treatment before transfer regardless of EAMT eligibility. Fourth, indirect costs were estimated based on the length of hospital stay, which may also result in underestimation of the true economic burden. Fifth, medical costs vary substantially across countries and healthcare systems, which limits the generalizability of our findings.

Lastly, the unequal sample sizes between the EAMT and non-EAMT groups may affect the comparability of outcomes. This imbalance reflects the urgent and selective nature of emergency evacuations, where patients often present with more severe conditions. As a result, residual confounding may remain despite statistical adjustments. Future studies should consider matched cohort or prospective study designs to enhance group comparability and improve causal interpretation.

## Conclusion

This study highlights the substantial economic burden associated with emergency air medical evacuations from remote islands, with cardiovascular diseases, injuries and fractures identified as major contributors. These findings underscore the importance of targeted preventive measures and improved healthcare accessibility to mitigate both the economic impact and health risks for individuals in remote island communities. Provide essential components for further cost-effective analysis of the dilemma between investment for faster and safer evacuation and implementation of more advanced facilities and medical personnels.

## Data Availability

The raw data supporting the conclusions of this article will be made available by the authors, without undue reservation. Patient data cannot be made publicly available due to privacy concerns. De-identified tabular data can be obtained from the corresponding author upon approval from the ethics committee of the Tri-Service General Hospital. Approval may be requested through the Tri-Service General Hospital’s Clinical Trial Management System (https://tsgh.cims.tw/wiPtms/index.html), with an expected review period of approximately 2–3 months. After approval, the raw data supporting the conclusions of this article will be made available by the authors.
