# Supplementary material for: Economic impact of patients with medical evacuation in remote islands: a case study in Matsu Islands
Source: Front Public Health. 2025 May 30;13:1542172. doi: 10.3389/fpubh.2025.1542172 (PMC12162960; doi:10.3389/fpubh.2025.1542172)
Supplement: Supplementary file 1 [file Table_1.docx]

**Supplement**

**Supplementary Table 1.** ICD-10 categories overview

| **Category** | **ICD-10 code** | **Description** |
| --- | --- | --- |
| Infectious | A00-B99 | Certain infectious and parasitic diseases |
| Oncology & Hematology | C00-D99 | Neoplasms; Diseases of the blood and blood-forming organs and certain disorders involving the immune mechanism |
| Endocrine | E00-E89 | Endocrine, nutritional, and metabolic diseases |
| Psychiatry | F01-F99 | Mental, behavioral and neurodevelopmental disorders |
| Neurologic | G00-G99 | Diseases of the nervous system |
| Eye &Ear | H00-H59 | Diseases of the eye and adnexa; Diseases of the ear and mastoid |
| Cardiovascular | I00-I99 | Diseases of the circulatory system |
| Respiratory | J00-J99 | Diseases of the respiratory system |
| Gastrointestinal | K00-K95 | Diseases of the digestive system |
| Skin | L00-L99 | Diseases of the skin and subcutaneous tissue |
| Musculoskeletal | M00-M99 | Diseases of the musculoskeletal system and connective tissue |
| Genitourinary | N00-N99 | Diseases of the genitourinary system |
| Obstetrics | O00-O9A | Pregnancy, childbirth, and the puerperium |
| Perinatal | P00-P96 | Certain conditions originating in the perinatal period |
| Congenital | Q00-Q99 | Congenital malformations, deformations, and chromosomal abnormalities |
| General conditions & others | R00-R99 | Symptoms, signs, and abnormal clinical and laboratory findings, not elsewhere classified |
| Injury | S00-T88 | Injury, poisoning, and certain other consequences of external causes |
| Accidents | V00-Y99 | External causes of morbidity |
| Health Service | Z00-Z99 | Factors influencing health status and contact with health services |

**Supplementary Table 2.** Number of patients by year

| **Year** | **2016** | **2017** | **2018** | **2019** | **2020** | **2021** | **2022** |
| --- | --- | --- | --- | --- | --- | --- | --- |
| EAMT | 11 | 12 | 29 | 19 | 24 | 25 | 16 |
| Non-EAMT | 23 | 32 | 44 | 44 | 72 | 60 | 12 |
| Total | 34 | 44 | 73 | 63 | 96 | 85 | 28 |

| Abbreviation: EAMT, Emergency Air Medical Transport |
| --- |

**Supplementary Table 3.** Number of patients according to ICD-10 classification

| **ICD-10 Code Definition** | **EAMT** | **Non-EAMT** |
| --- | --- | --- |
| **I (Certain infectious and parasitic diseases, A00~B99)** | | |
| A4 (Other bacterial diseases) | 3 | 13 |
| A8 (Viral infections of the central nervous system) | 0 | 1 |
| B1 (Viral hepatitis) | 0 | 1 |
| B2 (Human immunodeficiency virus [HIV] disease) | 0 | 1 |
| **II (Neoplasms, C00~D48)** | | |
| C1 (Malignant neoplasms of lip, oral cavity and pharynx) | 0 | 1 |
| C2 (Malignant neoplasms of digestive organs) | 3 | 2 |
| C3 (Malignant neoplasms of respiratory and intrathoracic organs) | 0 | 4 |
| C5 (Malignant neoplasm of breast and female genital organs) | 0 | 1 |
| C6 (Malignant neoplasms of male genital organs and urinary tract) | 0 | 1 |
| C8 (Different types of lymphoma) | 1 | 1 |
| D1, D2, D3 (Different types of benign Neoplasm) | 0 | 4 |
| D4 (Neoplasm of uncertain or unknown behavior) | 0 | 1 |
| **IV (Endocrine, nutritional and metabolic diseases, E00~E90)** | | |
| E0 (Disorders of thyroid gland) | 0 | 1 |
| E1 (Diabetes mellitus, or other disorders of glucose regulation and pancreatic internal secretion) | 0 | 1 |
| E8 (Metabolic disorders) | 0 | 1 |
| V (Mental and behavioral disorders, F00~F99) |  |  |
| F1 (Mental and behavioral disorders due to psychoactive substance use) | 0 | 1 |
| F3 (Mood [affective] disorders) | 0 | 1 |
| F9 (Behavioral and emotional disorders with onset usually occurring in childhood and adolescence) | 0 | 1 |
| **VI (Diseases of the nervous system, G00~G99)** | | |
| G4 (Episodic and paroxysmal disorders) | 2 | 2 |
| G5 (Nerve, nerve root and plexus disorders) | 0 | 2 |
| G8 (Cerebral palsy and other paralytic syndromes) | 1 | 0 |
| **VII (Diseases of the eye and adnexa, H00~H59)** | | |
| H0 (Disorders of eyelid, lacrimal system and orbit) | 0 | 1 |
| H1 (Disorders of conjunctiva, sclera, cornea, iris and ciliary body) | 0 | 2 |
| H4 (Glaucoma, Disorders of vitreous body and globe and Disorders of optic nerve and visual pathways) | 0 | 1 |
| H5 (Visual disturbances and blindness) | 0 | 1 |
| **VIII (Diseases of the ear and mastoid process, H60~H95)** | | |
| H8 (Diseases of inner ear) | 0 | 1 |
| H9 (Other disorders of ear) | 0 | 3 |
| **IX (Diseases of the circulatory system, I00~I99)** | | |
| I1 (Hypertensive diseases) | 0 | 3 |
| I2 (Ischemic heart diseases, Pulmonary heart disease and diseases of pulmonary circulation) | 15 | 6 |
| I4 (Disorders of cardiac myocytes and arrhythmia) | 4 | 1 |
| I5 (Heart failure) | 1 | 2 |
| I6 (Cerebrovascular diseases) | 15 | 8 |
| I7 (Diseases of arteries, arterioles and capillaries) | 2 | 0 |
| I8 (Diseases of veins, lymphatic vessels and lymph nodes, not elsewhere classified) | 0 | 1 |
| **X (Diseases of the respiratory system, J00~J99)** | | |
| J0 (Acute upper respiratory infections) | 0 | 2 |
| J1 (Influenza and pneumonia) | 4 | 8 |
| J3 (Other diseases of upper respiratory tract) | 0 | 1 |
| J4 (Chronic lower respiratory diseases) | 0 | 2 |
| J8 (Other respiratory diseases principally affecting the interstitium; Suppurative and necrotic conditions of lower respiratory tract) | 1 | 3 |
| J9 (Other diseases of pleura and other respiratory system) | 8 | 11 |
| **XI (Diseases of the digestive system, K00~K93)** | | |
| K2 (Diseases of esophagus, stomach and duodenum) | 2 | 2 |
| K3 (Diseases of appendix) | 0 | 12 |
| K5 (Noninfective enteritis and colitis; Other diseases of intestines) | 1 | 1 |
| K6 (Diseases of peritoneum) | 2 | 1 |
| K7 (Diseases of liver) | 0 | 2 |
| K8 (Disorders of gallbladder, biliary tract and pancreas) | 2 | 6 |
| K9 (Other diseases of the digestive system) | 1 | 3 |
| **XII (Diseases of the skin and subcutaneous tissue, L00~L99)** | | |
| L0 (Infections of the skin and subcutaneous tissue) | 1 | 4 |
| **XIII (Diseases of the musculoskeletal system and connective tissue, M00~M99)** | | |
| M0 (Infectious arthropathies) | 0 | 1 |
| M1 (Inflammatory polyarthropathies and arthrosis) | 2 | 0 |
| M2 (Other joint disorders) | 0 | 5 |
| M4 (Deforming dorsopathies and Spondylopathies) | 0 | 1 |
| M5 (Other dorsopathies) | 2 | 1 |
| M8 (Disorders of bone density and structure; Other osteopathies) | 6 | 2 |
| **XIV (Diseases of the genitourinary system, N00~N99)** | | |
| N1 (Renal tubulo-interstitial diseases; Renal failure) | 1 | 2 |
| N2 (Urolithiasis and Other disorders of kidney and ureter) | 0 | 2 |
| N3 (Other diseases of urinary system) | 1 | 3 |
| N4 (Diseases of male genital organs) | 0 | 1 |
| N7 (Inflammatory diseases of female pelvic organs) | 0 | 2 |
| **XV (Pregnancy, childbirth and the puerperium, O00~O99)** | | |
| O1 (Oedema, proteinuria and hypertensive disorders in pregnancy, childbirth and the puerperium) | 1 | 0 |
| O2 (Other maternal disorders predominantly related to pregnancy) | 0 | 1 |
| O6 (Complications of labor and delivery) | 1 | 1 |
| **XVIII (Symptoms, signs and abnormal clinical and laboratory findings, not elsewhere classified, R00~R99)** | | |
| R0 (Symptoms and signs involving the circulatory and respiratory systems) | 7 | 8 |
| R1 (Symptoms and signs involving the digestive system and abdomen) | 3 | 21 |
| R3 (Symptoms and signs involving the urinary system) | 0 | 4 |
| R4 (Symptoms and signs involving cognition, perception, emotional state, speech, voice and behavior) | 1 | 7 |
| R5 (General symptoms including fever, headache, pain, malaise, fatigue, senility, syncope, collapse, convulsions, shock, hemorrhage and enlarged lymph nodes) | 9 | 23 |
| R6 (General symptoms including oedema, hyperhidrosis, lack of expected normal physiological development, symptoms and signs concerning food and fluid intake, cachexia and systemic inflammatory response syndrome [SIRS]) | 1 | 2 |
| R9 (Abnormal findings on diagnostic imaging and in function studies, without diagnosis; Ill-defined and unknown causes of mortality) | 0 | 1 |
| **XIX (Injury, poisoning and certain other consequences of external causes, S00~T98)** | | |
| S0 (Injuries to the head) | 7 | 15 |
| S2 (Injuries to the thorax) | 0 | 3 |
| S3 (Injuries to the abdomen, lower back, lumbar spine and pelvis) | 4 | 2 |
| S4 (Injuries to the shoulder and upper arm) | 0 | 11 |
| S5 (Injuries to the elbow and forearm) | 1 | 6 |
| S6 (Injuries to the wrist and hand) | 1 | 11 |
| S7 (Injuries to the hip and thigh) | 9 | 3 |
| S8 (Injuries to the knee and lower leg) | 5 | 8 |
| S9 (Injuries to the ankle and foot) | 2 | 4 |
| T0 (Injuries involving multiple body regions) | 2 | 2 |
| T2 (Burns and corrosions) | 0 | 1 |
| **XXI (Factors influencing health status and contact with health services, Z00~Z99)** | | |
| Z1 (Special screening examination for infectious and parasitic diseases, neoplasms, and other diseases or disorders) | 0 | 2 |
| Z2 (Persons with potential health hazards related to communicable diseases) | 1 | 2 |

Abbreviation: ICD-10, International Statistical Classification of Diseases and Related Health Problems, 10th Revision; EAMT, Emergency Air Medical Transport

**Supplementary Table 4.** Results of the generalized linear regression model

| **Covariates** | **Coefficient** | **Std. Err** | | **p Value** | |
| --- | --- | --- | --- | --- | --- |
| **Sex** | | | | | |
| Male | 0.107 | | 0.113 | | 0.347 |
| Female | (Reference) | |  | |  |
| **Age group** |  | |  | |  |
| < 24 | (Reference) | |  | |  |
| 25 - 34 | 0.516 | | 0.181 | | 0.005** |
| 35 - 44 | 0.003 | | 0.193 | | 0.990 |
| 45 – 54 | 0.265 | | 0.166 | | 0.112 |
| 55 – 64 | 0.285 | | 0.170 | | 0.095 |
| > 65 | 0.288 | | 0.169 | | 0.089 |
| **EAMT** | | | | | |
| EAMT | 0.763 | | 0.104 | | <0.001*** |
| non-EAMT | Reference | |  | |  |

p-value: ‘***’ <0.001, ‘**’ <0.01, ‘*’ 0.05; EAMT, emergency air medical transport

**Appendix I.** Criteria for emergency air medical transport (EAMT)

According to the Administrative Regulations of Air Ambulance in Taiwan, the criteria for conducting emergency air medical transport are listed as follows:

**I. Basic Principles.**

1. When local medical resources are unable to provide treatment according to their equipment and specialties, and with urgency of time and medical condition, and where the patient’s life is in danger if not rescued by Emergency Air Medical Transport.
2. The hospital receiving the referral or treatment can provide timely and accurate medical treatment to the patient.
3. Adequate equipment and well-trained ambulance personnel are available to accompany the Emergency Air Medical Transport.

**II. Medical Indications:**

1. Revised Trauma Score (RTS) less than 12, or less than 5 years of age with a Pediatric Trauma Score (PTS) less than 9.
2. Glasgow Coma Scale (GCS) less than 10 or encountered more than 2 points decline.
3. Penetrating or crush injuries to the head, neck, or torso resulting in unstable vital signs.
4. Severe trauma or paralyzing of the spine or spinal cord.
5. Complete or incomplete amputations (excluding finger and toe amputations).
6. Two or more long bone fractures or severe pelvic fractures.
7. Second- or third-degree burns up to 10%, or burns to the face or perineum.
8. Drowning with severe respiratory complications.
9. Organ failure requiring aggressive treatment of intensive care.
10. Hypothermia requiring immediate and aggressive treatment (including invasive treatment).
11. Adult patients with a respiratory rate greater than 30 or less than 10 breaths per minute, heart rate greater than 150 or less than 50 beats per minute.
12. Cardiac chest pain, aortic dissection, aneurysm leaks, acute stroke, and seizures.
13. High-risk labors or newborns.
14. Other conditions that will affect the timeliness of emergency medical treatment if not rescued by emergency air medical transport.
